# Analysis of the loss of Epithelial Cadherin expression in gastric cancer in Zambia

**Published:** 2025-02-06

**Authors:** Vutisa Dokowe, Mupeta Songwe, Chibamba Mumba, Violet Kayamba

**Affiliations:** 1 University Teaching Hospital, Department of Pathology and Microbiology, Nationalist Road, Private Bag RW IX, Lusaka, Zambia; 2 University of Zambia, School of Medicine, Nationalist Road, PO Box 50110, Lusaka, Zambia; 3 Tropical Gastroenterology and Nutrition Group, 26 Esther Lungu Road, Kamwala South, Lusaka, Zambia

**Keywords:** Gastric cancer, E-cadherin, Zambia

## Abstract

**Background:**

Gastric cancer (GC) is the one of the most common cancers globally and can be classified as diffuse, intestinal or mixed. Epithelial-cadherin (E-cadherin) is a transmembrane glycoprotein which plays a role in maintaining cell shape and extracellular matrix and its loss or reduction has been observed in gastric carcinogenesis. The aim of the study was to determine the proportion of loss of E-cadherin expression in GC.

**Methods:**

Archival formalin fixed paraffin embedded GC tissues and their basic characteristics were retrieved from the histopathology laboratory at the University Teaching Hospital in Lusaka. Sections of the tissue were stained with haematoxylin and eosin to allow for typing of the GC and immunohistochemically stained with an antibody against E-cadherin. Data analysis was conducted using Stata version 15.

**Results:**

We successfully stained 31 archival GC tissues after excluding those not meeting quality checks. Among the samples included, 18 (58%) were from females. The median age was 51 years, IQR 40–68. Overall, 11 (36%) showed loss of E-cadherin expression. Young age was associated with E-cadherin loss (p=0.02). Histologically, 10 (32%) of the GC were of the diffuse type, 18 (58%) intestinal type and 3 (10%) mixed type. E-cadherin loss was significantly higher in diffuse type 7 (70%) than the Intestinal 2 (18%) or 2 (67%) the mixed types (p=0.003). In multivariable analysis, the difference remained significant (p=0.041). The proportion of E-cadherin loss was higher in tumours located in the cardia, 57%, when compared to those that were non-cardia in location, 29%, but the difference was not statistically significant [OR 3.2; 95% CI 0.4–27, p=0.21].

**Conclusions:**

Loss of E-cadherin expression is associated with a younger age at diagnosis and with diffuse type gastric cancer. Results from this study could contribute toward patient prognostication and possibly influence therapeutic choices for GC patients in Zambia.

## INTRODUCTION

Gastric cancer (GC) is the fifth most common cancer globally, with 968,784 cases reported in 2022^1^. In Zambia, GC ranks ninth among all cancers with current evidence that it will more than double by 2040.^1^ Clinical outcomes for GC are very poor mostly due to late diagnosis.^2,3^ The aetiology of GC is multifactorial, involving both environmental and genetic influences.^4,5^ The progression of the disease is complex and can occur decades after exposure to carcinogenic agents, highlighting its heterogeneous nature and ongoing relevance as a global health issue.^5^ Histologically, GC can be classified according to Lauren’s criteria, which identifies three types: diffuse, intestinal, and mixed, each exhibiting distinct histomorphological characteristics.^7^

Central to the development of GC is E-cadherin, a transmembrane glycoprotein encoded by the CDH1 gene.^8^ E-cadherin plays a crucial role in maintaining cell adhesion and tissue integrity, acting as a fundamental tumour suppressor that mediates contact inhibition of cellular proliferation.^9^ The loss of E-cadherin expression contributes to the epithelial-mesenchymal transition (EMT), a critical process that enhances cell motility, invasion, and the metastatic potential of cancer cells.^10,11^ Furthermore, EMT facilitates the acquisition of cancer stem cell characteristics, which can lead to increased drug resistance and hinder the effectiveness of treatment.^12^ Decreased E-cadherin expression in GC tissues is correlated with higher malignancy grades and poorer prognoses.^13^ As research continues to explore novel treatment modalities, understanding the prevalence of E-cadherin loss in GC patients is crucial for improving survival outcomes, particularly in advanced cases.^14^

The justification for this study arose from the significant gap in knowledge regarding the proportion of GC patients in Zambia who exhibit loss of E-cadherin expression. E-cadherin is recognized as an important prognostic marker that facilitates improved risk stratification, allowing for more tailored treatment approaches for patients with GC.^15^ To our knowledge, there are no previous studies having looked at E-cadherin expression in Zambia.

The aim of this study was to generate critical data on the prevalence of E-cadherin loss by establishing the proportion of patients affected. To guide cancer precision therapy, knowing the molecular characteristics is vital, and this study provides some baseline information on this subject. In addition, our findings will contribute valuable insights that can inform clinical guidelines and enhance understanding of molecular characteristics of GC.

## METHODS

### Study Design

This was a pilot cross-sectional study using archival formalin fixed paraffin embedded (FFPE) tissue blocks from patients diagnosed with GC to assess loss of expression of E-cadherin using immunohistochemistry.

### Study Population and data source

The study was conducted at the University Teaching Hospital (UTH) adult hospital, Department of Pathology and Microbiology, in Lusaka, Zambia.

The study population included all GC cases diagnosed at UTH from January 2021 to December 2023. Patients were identified and their relevant clinical information was retrieved from the Data Intensive System and Application (DISA) and from clinical requisition records and endoscopy reports stored within the department. The variables included sex, age, residence, tumour topography, indication for endoscopy and histologic type by Lauren classification. Using consecutive sampling, all cases with histological diagnosis of GC were considered. Excluded, were those with inadequate or poorly preserved tissue and missing clinical details. Ethical clearance was obtained from the University of Zambia Biomedical Ethics Committee, Ref no. 4219–2023.

### Data collection and specimen processing

Data in the UTH histopathology laboratory is collected using the DISA system. We searched for cases in this system using the key words; ‘gastric’, ‘gastric cancer’ and ‘gastric adenocarcinoma’. Unique identity number of retrieved reports were used to identify and collect the archival FFPE blocks. Additional data was obtained from the endoscopy reports.

**Figure 1:**
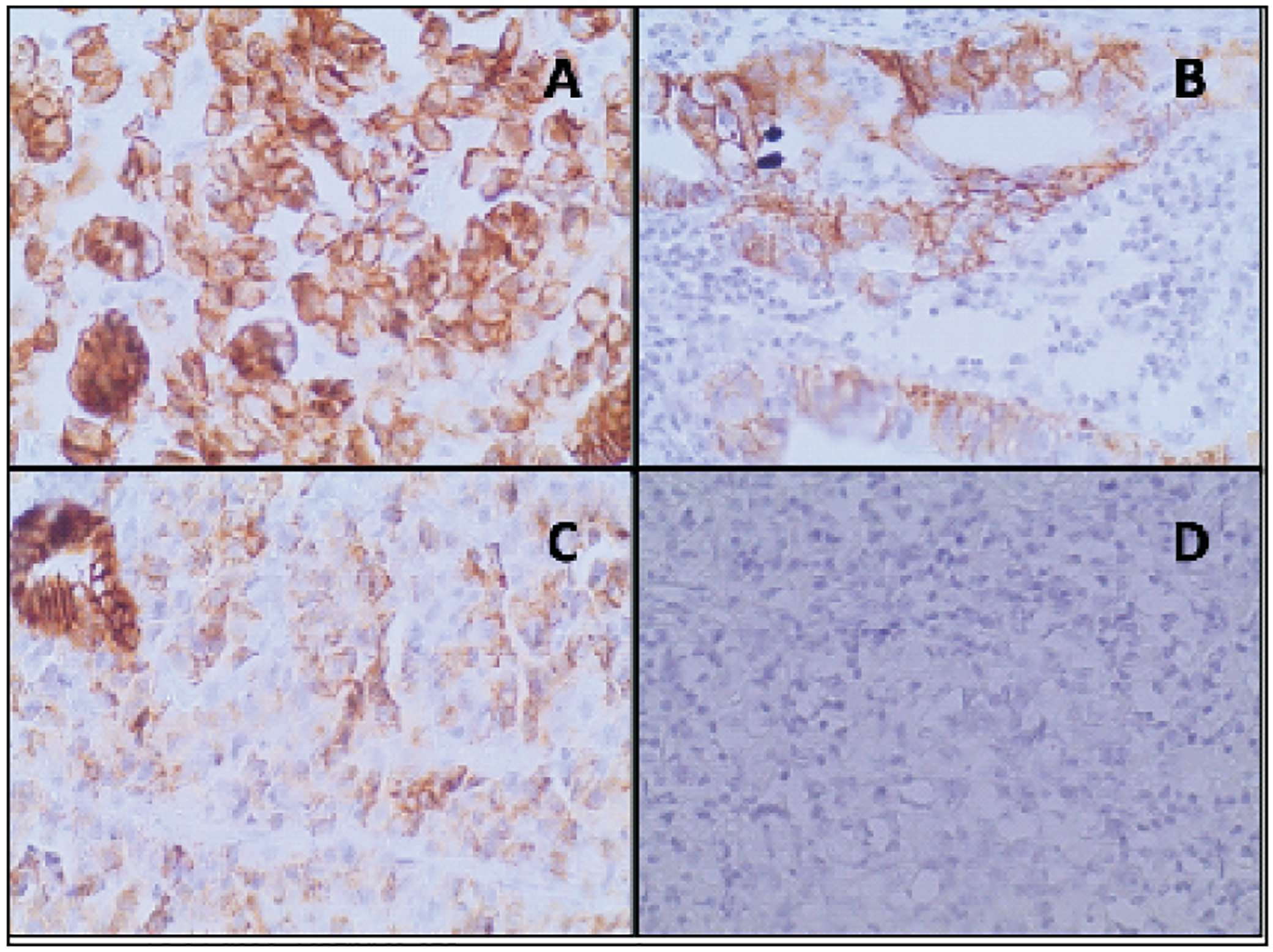
Histological images for E-cadherin staining, at X400 (A)Positive staining for diffuse type gastric cancer, (B) Positive staining for intestinal type gastric cancer, (C) Positive staining for mixed type gastric cancer and (D) Negative staining

Slides were processed according to the standard operating procedure (SOP) at the UTHs’ Histopathology laboratory for haematoxylin and eosin (H & E) staining. E-cadherin immunohistochemistry staining was done using Monoclonal Mouse, anti-Human E-cadherin, Agilent Technologies, Inc, USA, with strict adherence to the manufacturer’s instructions. All runs were conducted with positive and negative controls for result validation. Figure 1 shows examples of images obtained.

The slides were examined under the microscope by VD, and then later submitted to trained pathologists, SM and CM who reviewed them independently. Cohen’s Kappa statistics was used to analyse the inter-observer agreement between the two consultant pathologists and between the pathologists and VD. Any discrepancies were resolved by consensus. Examination of the slides was done using an Olympus CX 31 binocular biological microscope at the following magnification powers: X40, X100 and X400 to evaluate for E-cadherin expression. The intensity of the immunostaining was done using a four-tiered scale (negative = 0, weak = 1+, moderate = 2+ and strong = 3+). Grade 0 represented cases with no detectable immunostaining of tumour cells, whereas cases that were graded as 1+ exhibited weak staining of the majority of tumour cells. Tissue sections with a moderate or strong staining intensity were scored 2+ or 3+, respectively. In the final score, samples that had a moderate or strong immunostaining (score 2+ and 3+) were defined as ‘positive’, and samples that had a weak or absent immunostaining (score 0 and 1+) were defined as ‘negative’.

### Data analysis

The data were coded and imported into Stata 15 statistical software for analysis. For categorical variables, frequencies, proportions and percentages were used. For continuous variables, the median with interquartile ranges were computed. For associations, Fisher’s exact test was used for categorical variables and Kruskal Wallis (more than two) tests was used for continuous variables. A stepwise logistic regression to assess the associations was used while correcting for confounders. A 95% confidence interval with a two-sided p-value of <0.05 were considered statistically significant in all cases. As this was a pilot study, we included all samples with acceptable quality for IHC determination.

## RESULTS

We retrieved 141 FFPE tissue blocks. Of these, 98 had inadequate tissue, 7 had extensively crushed or poorly processed tissue, and 5 had missing clinical details. We were therefore able to stain, to acceptable quality, 31 samples, and these were included in the final analysis.

Overall, 11/31 (35%) of the cases showed loss of E-cadherin stain and 20/31 (65%) had positive staining. Of the cases included, 18 (58%) were female and 13 (42%) were male. The age range of the cases was between 23 and 76 years with a median of 51 years, IQR 40–68. Analysis of sex, age and residence by E-Cadherin status is as depicted in Table 1.

**Figure 2:**
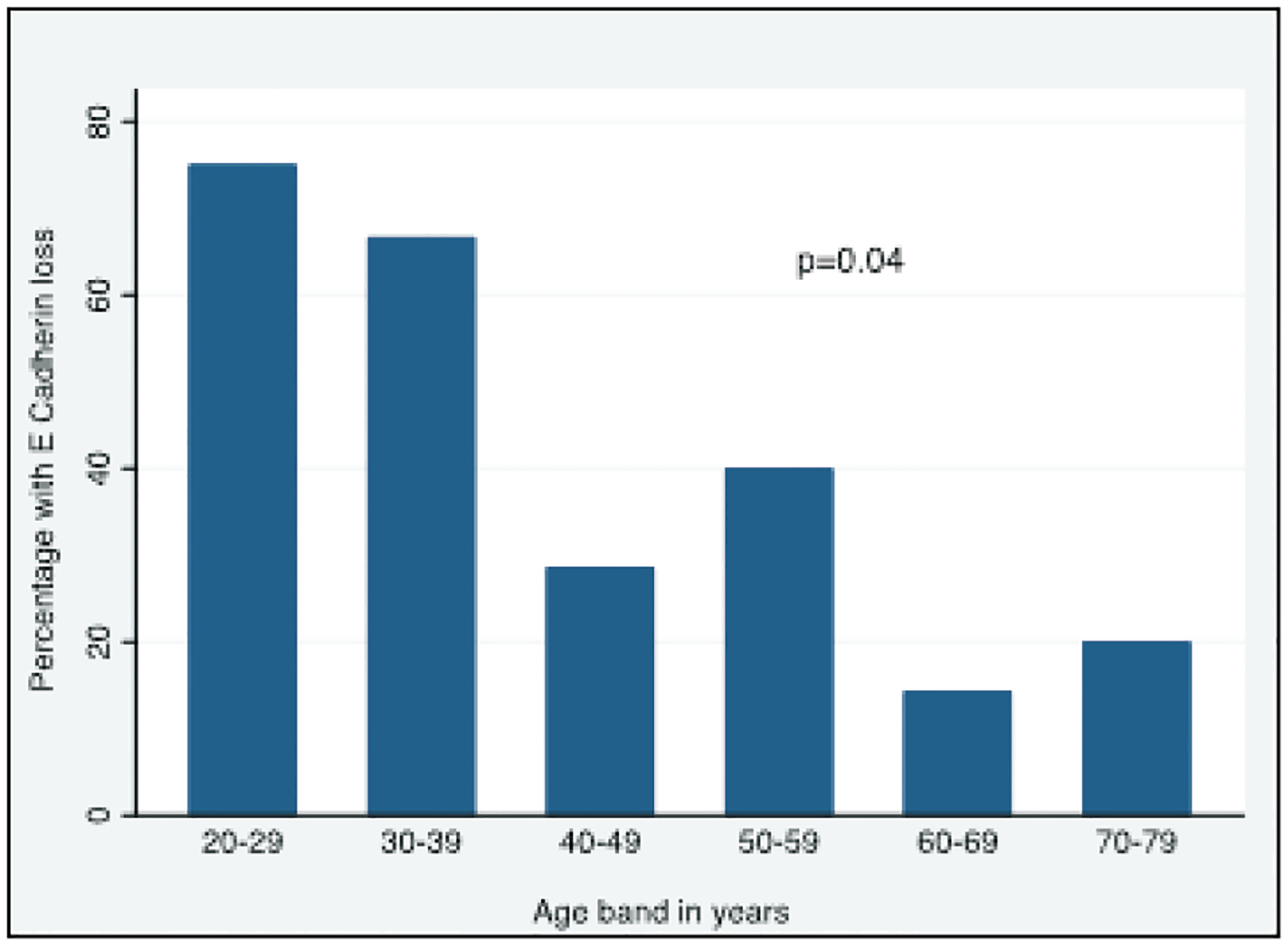
E-Cadherin expression in relation to 10-year age bands

We analysed loss of E-cadherin in relation of patient age. There were four patients in the 20–29 age band, three in the 30–39 age band, seven in the 40–49 age band, five in the 50–59 age band, seven in the 60–69 age band and 5 in the 70–79 age band, Figure 2. The proportion of E-cadherin loss was significantly higher in younger age bands than in the older ones, p=0.04.

Histologic classification showed that 10 (32%) were of the diffuse type, 18 (58%) intestinal type and 3 (10%) mixed type of GC. E-cadherin loss was significantly higher in diffuse type 7 (70%) than the Intestinal 2 (18%) or 2 (67%) of the mixed types (p=0.003), Table 2.

Anatomically, 7 (23%) of the tumours were located in the cardia and 24 (77%) were non-cardia. The proportion of E-cadherin loss was 57% among the cardia tumours and 29% among the non-cardia ones. The difference was not statistically significant [OR 3.2; 95% CI 0.4–27, p=0.21].

We ran a stepwise logistic regression that included female sex, age, Lusaka residence, tumour location and histological tumour type. Significant associations were found for young age, non-cardia tumour topography [OR 0.06, 95% CI 0.004–0.9, p=0.041] and histological tumour type [OR 0.04, 95% CI 0.001–0.9, p=0.041].

The inter-observer agreement for E-cadherin expression between the two pathologists was 71%, corresponding to a Kappa statistic of 0.52, indicating moderate agreement. The agreement between the first pathologist and the principal investigator was higher, at 83%, with a Kappa statistic of 0.72, reflecting substantial agreement. In contrast, the agreement between the second pathologist and the principal investigator was 70%, with a Kappa statistic of 0.52, indicating moderate agreement as well. All discrepancies in results were thoroughly discussed among the evaluators, leading to a consensus that determined the final reported results.

## DISCUSSION

This study aimed to evaluate the loss of E-cadherin expression in GC cases in Zambia, revealing a significant proportion of loss of expression. This finding is noteworthy, as E-cadherin plays a crucial role in maintaining cellular adhesion and suppressing tumour progression. It is an important component of the adherens junction and hemidesmosomes playing a critical role in maintaining cell shape, polarity and cell differentiation. Our study further identified significant associations between loss of E-cadherin expression and age, tumour location and histomorphology. Loss of E-cadherin expression was higher in the diffuse sub-type of GC.

E-cadherin is integral to the integrity of epithelial tissues, acting as a tumour suppressor by mediating contact inhibition and preventing excessive cell proliferation. As noted, the loss of E-cadherin is often correlated with poorly differentiated GC, which exhibit higher invasive potential and poor prognoses.^16,17^ E-cadherin expression in precancerous lesions (i.e. gastric atrophy, intestinal metaplasia and dysplasia) is not well studied as many studies that looked at this subject were are small.^18^ In one study, 40 gastrectomy specimens of patients with GC were evaluated.. E-cadherin loss was 9% in chronic atrophic gastritis and 57% in intestinal metaplasia. They concluded that E-cadherin loss was observed in precancerous lesions and had potential for diagnostic and therapeutic implications.^18^ In another study, 163 formalin fixed paraffin embedded blocks with gastric carcinoma were evaluated. Of these, 44 had gastric mucosal dysplasia, 25 with intestinal metaplasia, 28 with atrophic gastritis and 12 were healthy controls. Intestinal metaplasia, atrophic gastritis and control biopsy specimens had normal membrane staining for E-cadherin. They reported that 36% with gastric dysplasia stained abnormally for E-cadherin and abnormal expression was demonstrated in 46% of gastric carcinoma. They concluded that abnormal expression of E-cadherin occurred frequently in gastric carcinoma.^19^

Our results align with existing literature indicating a trend towards increased E-cadherin loss in younger patients. This is consistent with findings from Schildberg et al., who noted a similar age demographic in families with hereditary diffuse GC (HDGC).^20^ While our study did not categorize the patients into those with hereditary cancer and those with sporadic forms, the shared age range warrants further investigation into the molecular underpinnings and potential genetic predispositions associated with early-onset gastric cancer.

Moreover, we observed a significant association between E-cadherin loss and tumour location, particularly in the upper regions of the stomach (cardia and fundus). This is in contrast to with the analysis by Prathipa et al., which highlighted that while the antrum was a common site for GC, the relationship between E-cadherin expression and tumour location remains complex.^21^

In our study, we also confirmed the association between E-cadherin loss and diffuse-type GC, echoing findings from Ascano et al. that reported significant E-cadherin loss in diffuse cases.^22^ However, some diffuse-type cases in our analysis did not show loss of expression, which reflects the heterogeneity observed in GC and highlights the need for more nuanced investigations into the molecular variations that may underpin these discrepancies. Contrary to findings in some other studies, we did not find associations between E-cadherin loss and factors such as sex or residency, a result consistent across various demographics and locations. This reinforces the notion that E-cadherin dysfunction may act independently of these variables in gastric cancer pathogenesis.

The implications of E-cadherin loss extend beyond tumour biology into clinical outcomes. The disruption of E-cadherin-mediated cell adhesion facilitates epithelial-mesenchymal transition (EMT), leading to increased cell motility and a propensity for metastasis, ultimately resulting in poor prognosis.

Overall, the findings from this pilot study emphasize the importance of E-cadherin as a key biomarker in GC. In Zambia, the diagnosis of GC is a challenge as many health centres do not have the capability to conduct endoscopies. In addition, the number of trained endoscopists in the country is low. Understanding the molecular characteristics of GC in Zambia is slowly contributing towards the development of biomarkers that might be used in future to identify individuals at high risk. Previously, our group analysed other molecular features of GC in Zambia including HER2, MLH1, Epstein -Barr associated and more recently PD-L1 and CDX2 expression. All this work is contributing to our general understanding of GC in Zambia.

Despite the small sample size, the significant associations with age, tumour location, and histological type underscore the need for larger, more comprehensive studies to further elucidate the role of E-cadherin in gastric cancer pathogenesis and its potential as a therapeutic target. Given the poor prognosis for newly diagnosed gastric cancer in Zambia.^2^ Understanding these molecular mechanisms may ultimately guide better clinical management and improve outcomes for patients.

## LIMITATIONS

This study had several limitations that may affect the generalizability and reliability of its findings. A significant challenge arose from the retrieval of tissue blocks from archives, which were poorly organized, complicating the identification of suitable samples. Additionally, instances of missing blocks reduced the overall sample size available for analysis. Some tissue blocks had been sent for molecular testing by other facilities, further limiting the samples accessible for our research. Among those obtained, many contained insufficient or no tissue, preventing a definitive diagnosis of gastric cancer.

Moreover, several blocks displayed extensive crush artifacts, compromising tissue integrity and making accurate diagnoses impossible. Incomplete clinical details for some cases also hindered their inclusion in the study, potentially introducing bias and limiting the comprehensiveness of our analysis. These limitations highlight the need for improved sample management and documentation in future research to enhance the reliability and validity of findings in gastric cancer studies. Addressing these challenges will be essential for obtaining more robust and informative results moving forward.

## CONCLUSION

In conclusion, this pilot study reveals a significant loss of E-cadherin expression in younger ages, proximal tumour locations, and diffuse sub-type of GC. Future research is warranted to investigate the molecular mechanisms underlying E-cadherin loss, particularly regarding genetic predispositions in younger patients and its relationship with tumour behaviour. This study was limited by the small sample size. Larger, multi-centre studies, probably using genetic sequencing, are necessary to validate these findings and explore the therapeutic implications of targeting E-cadherin, considering its dual role in tumour progression.

## Figures and Tables

**Table 1:** Basic clinicopathological characteristics of samples included in the final analysis

	E-cadherin expression			
Variable	Positive N (%)	Negative N (%)	OR (95% CI)	p-value
**Sex:**				
Male	11 (55)	7 (64)	0.7 (0.1–3.9)	0.72
Female	9 (45)	4 (36)		
**Age in years (IQR)**	58 (43–69)	40 (27–56)	-	0.02
**Tumour Topography:**			-	
Cardia	3 (15)	4 (36)		0.06
Fundus	2 (10)	2 (18)		
Body	8 (40)	4 (36)		
Antrum	7 (35)	1 (10)		
**Lauren classification:**			-	0.07
Diffuse	3 (15)	7 (64)		
Intestinal	16 (80)	2 (18)		
Mixed	1 (5)	2 (18)		

* For binary variables, the odds ratios and p-values were computed using Fisher’s exact test

* For variables with more than two categories, p-values were computed using the Kruskal-Wallis test

**Table 2:** Evaluation of associations between loss of E-Cadherin expression and clinicopathological characteristics

	Univariate analysis		Multivariable analysis	
	Using Fisher’s exact and the Kruskal-Wallis		Using stepwise logistic regression	
Variable	OR (95% CI)	p-value	OR (95% CI)	p-value
Female	0.7 (0.1–3.9)	0.72		
Age (as a continuous variable)	-	0.02	0.8 (0.7–0.98)	0.036
Lusaka residence	0.5 (0.1–2.7)	0.45		
Tumour location		0.06	0.06 (0.004–0.9)	0.041
Histological type of tumour (Lauren classification)	-	0.03	0.04 (0.001–0.9)	0.041

## What is already known on this topic:

- Gastric cancer is one of the leading causes of cancer related morbidity and mortality globally, but in Zambia it is poorly investigated
- Diffuse type of gastric cancer has a poorer prognosis and is associated with familial syndromes

## What this study adds:

- This study has shown that gastric cancer with loss of E-cadherin expression is higher in in younger ages and proximally located tumours
- It has also confirmed that loss of E-cadherin expression is associated with diffuse sub-type of gastric cancer
